# Crystal structure and Hirshfeld surface analysis of poly[tris­(μ_4_-benzene-1,4-di­carboxyl­ato)tetra­kis­(di­methyl­formamide)­trinickel(II)]: a two-dimensional coordination network

**DOI:** 10.1107/S2056989019014658

**Published:** 2019-11-08

**Authors:** Cesario Ajpi, Leopoldo Suescun, Naviana Leiva, Anders Lundblad, Göran Lindbergh, Saul Cabrera

**Affiliations:** a Departament of Inorganic Chemistry and Materials Science/Advanced Materials, IIQ Chemical Research Institute, UMSA Universidad Mayor de San Andres, La Paz, Bolivia; b Cryssmat-Lab/DETEMA, Facultad de Quimica, Universidad de la Republica, Montevideo, Uruguay; c Division of Safety and Transport/Electronics, RISE, Research Institutes of Sweden, SE-50462 Borås, Sweden; d Department of Chemical Engineering, Applied Electrochemistry, KTH Royal Institute of Technology, SE-100 44 Stockholm, Sweden

**Keywords:** crystal structure, Ni metal-organic coordination network, terephthalate, layered structure

## Abstract

The crystal structure of the title compound, [Ni_3_(C_8_H_4_O_4_)_3_(C_3_H_7_NO)_4_], is a two-dimensional coordination network formed by trinuclear linear Ni_3_(tp)_3_(DMF)_4_ units (tp = terephthalate = benzene-1,4-di­carboxyl­ate and DMF = di­methyl­formamide) displaying a characteristic coordination mode of acetate groups in polynuclear metal–organic compounds. Individual trinuclear units are connected through tp anions in a triangular network that forms layers. One of the DMF ligands points outwards and provides inter­actions with equivalent planes above and below, leaving the second ligand in a structural void much larger than the DMF mol­ecule, which shows positional disorder. Parallel planes are connected mainly through weak C—H⋯O, H⋯H and H⋯C inter­actions between DMF mol­ecules, as shown by Hirshfeld surface analysis.

## Abstract 2

The structure of *catena*-[tris­(μ_4_-benzene-1,4-di­carboxyl­ato)-tetra­kis­(μ_1_– di­methyl­formamide-κ^1^ *O*)-trinickel(II)], C_36_H_40_N_4_Ni_3_O_16_, has been determined in the monoclinic *P*2_1_/*n* space group. The compound has a two-dimensional coordination network structure and it is of inter­est with respect to lithium-ion battery applications. Hirshfeld surface analysis was performed to characterize inter­planar inter­actions. The structure exhibits disorder of coordinated solvent mol­ecules.

## Chemical context   

Extended hybrid organic–inorganic materials composed by transition metals and bridging carboxyl­ates are inter­esting compounds that include the well-known metal–organic frameworks (MOFs), coordination polymers (CP) and coord­ination networks (CN) (Batten *et al.*, 2013 ▸). In the last decade, much of the research into this kind of compounds has focused in the design of materials looking for tunability of potential industrial applications such as lithium-ion batteries (Shin *et al.*, 2015 ▸; Maiti *et al.*, 2015 ▸; Tian *et al.*, 2016 ▸), substitutes for dye-sensitized solar cells (DSSCs) (Zhang *et al.*, 2018 ▸; Yan *et al.*, 2018 ▸; Jeevadason *et al.*, 2014 ▸), luminescent compounds (Kara *et al.*, 2018 ▸; Igoa *et al.*, 2019 ▸) and magnetic materials (Mesbah *et al.*, 2014 ▸) among others. In the search for new extended hybrid materials based on Ni and terephthalate (terephthalate = tp = benzene-1,4-di­carboxyl­ate), the title compound [Ni_3_(C_8_H_4_O_4_)_3_(C_3_H_7_NO)_4_] was synthesized by a solvothermal process in di­methyl­formamide (DMF) and is currently under study for application as an anode material in lithium-ion batteries. In order to perform an adequate structure–property correlation, the crystal structure of the compound was determined and supra­molecular features of potential inter­est for understanding Li-ion inter­calation and migration were analysed using the Hirshfeld surface (HS).
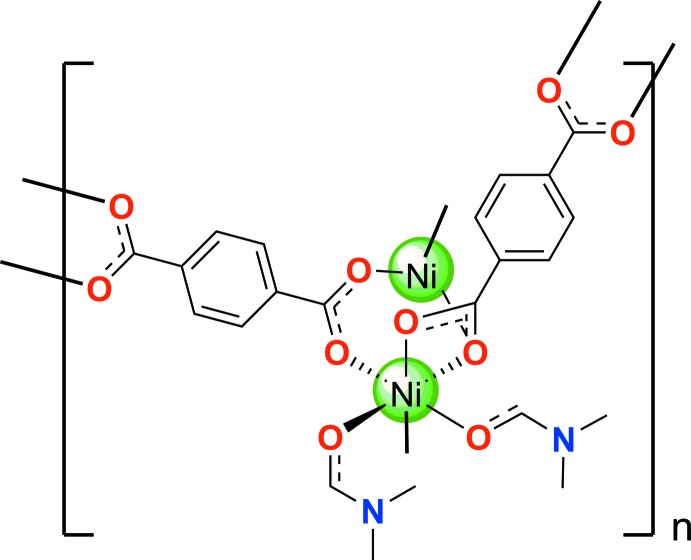


## Structural commentary   

**Figure 1:**
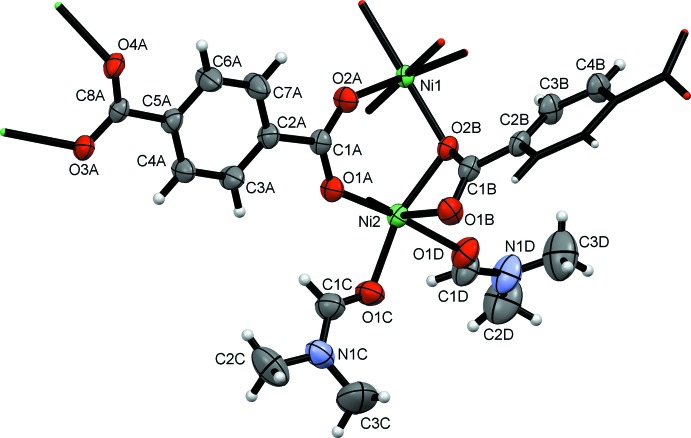
*ORTEP* model view of the asymmetric unit of [Ni_3_(C_8_H_4_O_4_)_3_(C_3_H_7_NO)_4_]. Displacement ellipsoids are represented at the 50% probability level. Atoms completing the connectivity of those in the asymmetric unit and half symmetry-equivalent tp anion are shown as coloured spheres of arbitrary radii. Only one position of the disordered DMF ligand is shown for clarity.

**Figure 2:**
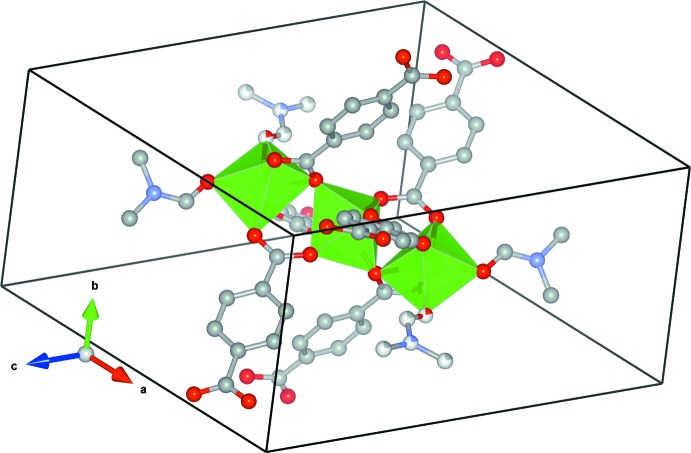
One trinuclear unit of [Ni_3_(C_8_H_4_O_4_)_3_(C_3_H_7_NO)_4_] highlighting the coordination polyhedra around each Ni atom and the coordination modes of the tp anions. Only one position of the disordered DMF ligand is shown and H atoms are omitted for clarity.

The title compound is a two-dimensional coordination polymer formed by linear trinuclear centrosymmetric Ni_3_(tp)_3_(DMF)_4_ units connected through tp anions, which crystallizes in the monoclinic *P*2_1_/*c* space group. Two distinct hexa­coordinated Ni^2+^ cations (Ni1 in a special position with occupancy factor 0.5), two DMF ligands and two tp anions (anion *B* in a special position with occupancy factor 0.5) exist in the asymmetric unit (Fig. 1 ▸). The central Ni atom, located on an inversion centre, displays an octa­hedral coord­ination to O atoms from three pairs of carboxyl­ate units belonging to three symmetry-related tp anions with Ni1—O bond distances in the range 2.0205 (14)–2.0868 (14) Å and a maximum deviation of 4.85° from the expected O—Ni1—O octa­hedral bond angles. The two terminal Ni2 cations also coordinate the carboxyl­ate units of three symmetry-related tp units, one of them in bidentate mode, and two independent di­methyl­formamide ligands (one of them showing positional disorder) in a significantly distorted octa­hedron (Fig. 2 ▸). Ni2—O bond distances are in the range 2.0090 (15)–2.0791 (15) Å for terephthalate and 2.042 (12)–2.1853 (16) Å for DMF oxygen atoms respectively (including the lower occupancy disordered ligands). The O1*B*—Ni2—O2*B* angle of 61.52 (6)° corresponding to a tridentate carboxyl­ate, acting as bidentate towards Ni2, is very far away for the expected octa­hedral 90° angle. However, the coord­in­ation is still octa­hedral since O1*B*, O2*B*, O1*C* and O3*A* form a clear equatorial plane with Ni deviating by just 0.1202 (7) Å from the plane and the rest of the equatorial bond angles [O2*B*—Ni2—O3*A =* 99.18 (6), O3*A*—Ni2—O1*C* = 99.13 (7) and O1*C*—Ni2—O1*B* = 98.91 (7)°] are increased by about 10° to compensate for the very small angle from the bidentate ligand (O3*A* is in position 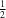 + *x*, 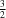 − *y*, 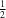 + *z*). Additionally the two apical atoms O1*A* and O1*D* lie 2.026 (6) and 2.1269 (16) Å, respectively, from the equatorial plane, forming an O1*BD*—Ni2—O1*A* angle of 176.0 (6)°. The carboxyl­ate that is bidentate towards Ni2 is also monodentate towards Ni1, with the O2*B* atom being the link between corner-sharing Ni1 and Ni2 octa­hedra, which explains the longer Ni—O2*B* bond distances of 2.0868 (14) and 2.0791 (15) Å to Ni1 and Ni2 respectively, compared with all other Ni—O_tp_ bond distances (see Fig. 2 ▸). The trinuclear octa­hedral arrangement with the three Ni atoms coordinated exclusively by O has only been observed in one 1,3-benzene­dicarboxyl­ate *catena*-[bis­(μ_4_-isophthalato)bis­(μ_3_-isophthalato)trinickel(II) bis­(3-ethyl-1-methyl-1*H*-imidazol-3-ium)] (Chen *et al.*, 2011 ▸) where the Ni cations are connected through the same number and coordination modes of carboxyl­ate moieties. In that compound, however, two additional carboxyl­ates complete the coordination spheres of the terminal Ni cations, instead of DMF mol­ecules, giving a three-dimensional connected network. Ni1⋯Ni2 distances of 3.4414 (4) Å are observed, also found in the 1,2-benzene­dicarboxyl­ate (Ni_central_⋯Ni_terminal_ = 3.442 Å). This coordination mode is frequently found in other trinuclear transition metal carboxyl­ates, with and without different ligands bonded to the terminal cations.

**Figure 3:**
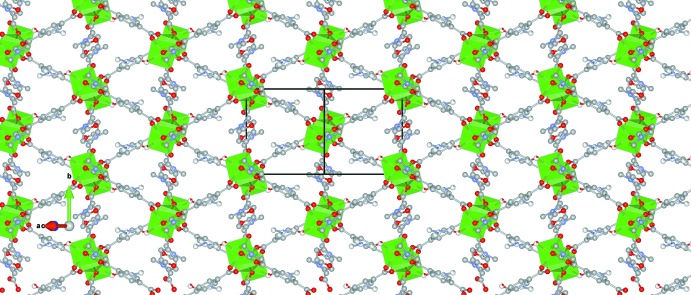
One plane of [Ni_3_(C_8_H_4_O_4_)_3_(C_3_H_7_NO)_4_] showing the hexa­gonal arrangement of equivalent units with slightly distorted distances. The tilt of the trinuclear octa­hedral units is also visible. H atoms and disordered positions of the DMF ligand have been omitted for clarity.

Each terephthalate ion links two nearby trinuclear units forming a slightly distorted two-dimensional hexa­gonal arrangement along the crystallographic (10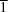) plane as shown in Fig. 3 ▸. Since the central Ni atom (Ni1) of the trinuclear arrangement is located at (0, 0, 0) and equivalent (½, ½, ½) coordinates, the hexa­gonal arrangement shows a 2 + 4 distance pattern with two opposite nearby units at 9.6335 (11) Å (equal to the *b*-axis length) and four at 10.1407 (9) Å (equal to half of the short body diagonal of the unit cell) defining isosceles triangles with one small [56.718 (8)°] and two larger [(61.641 (4)°] angles. The tp anions link nearby units in two different modes. The longest inter­unit distance corresponds to tp anions connecting the top or bottom parts of the unit, parallel to the plane (terephthalate anion *A*), while the shorter distance corresponds to a tp unit that is located over a centre of symmetry (anion *B*), which connects the top/bottom part of one unit to the bottom/top part of the next unit. This diagonal connection produces a tilt in the linear trinuclear units that are rotated by 11.82° from the normal to the plane of the network, in a direction slightly away from the *b* axis.

The ordered DMF mol­ecules (labelled *C*) point outwards at both sides of the planes providing a polar surface that allows for the inter­action of parallel planes of the coordination polymer. The disordered DMF ligands (labelled *D*) occupy part of the void space between consecutive planes (see Section 3) and were modelled over three different positions rotated by 180° and displaced respectively, which strongly suggests that both static and dynamic disorder are present.

## Supra­molecular features and Hirshfeld surface analysis   

**Figure 4:**
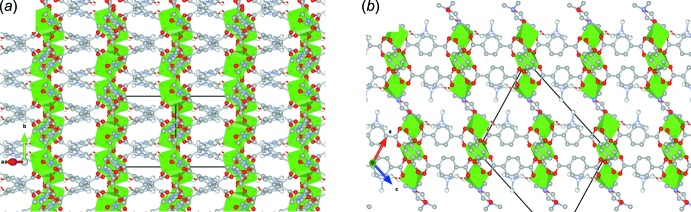
(*a*) View of two planes of the [Ni_3_(C_8_H_4_O_4_)_3_(C_3_H_7_NO)_4_] coordination polymer along [10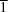] showing that the projection of one plane is shifted by *b*/2 with respect to the next one and (*b*) the same two planes projected along [010] showing the relative position of ordered and disordered DMF mol­ecules with respect to the planes.

Parallel planes do not stack in a typical hexa­gonal arrangement, where a layer projects over the voids of the poly[tris­(μ_4_-benzene-1,4-di­carboxyl­ato)tetra­kis­(di­methyl­formamide)­trinickel(II)], but in this case one layer projects over the center of the short inter-unit distance of the next layer, or is shifted by half of the *b*-axis length. This is again a consequence of the position of the Ni1 atoms at the corners and the centre of the unit cell, forming planes along (10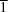). Fig. 4 ▸ *a* shows two parallel planes along the [10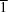] direction (compare with Fig. 3 ▸) where it is shown that the projection of one plane falls away from the voids in the next one. Fig. 4 ▸ *b* shows the same two planes along the [010] direction where the relative position of the ordered DMF ligands in consecutive layers is shown.

**Figure 5:**
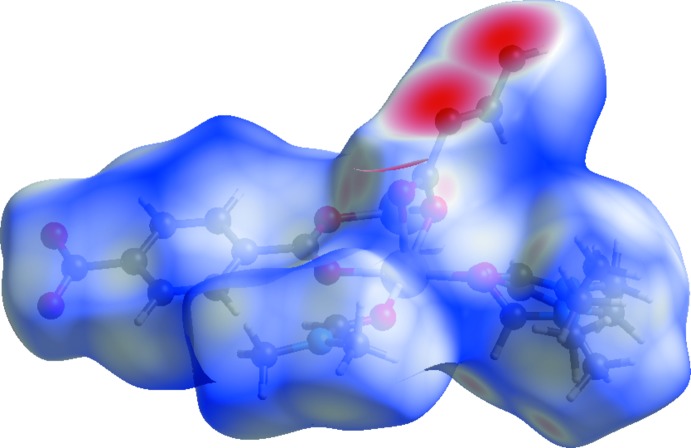
View of the three-dimensional Hirshfeld surface of the title complex plotted over *d_norm_* in the range −0.7548 to 1.5398 a.u.

**Figure 6:**
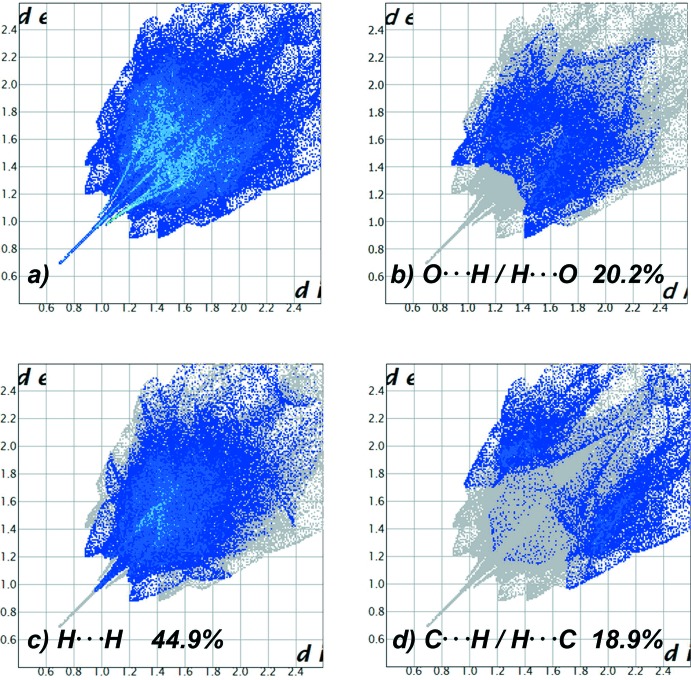
Two-dimensional fingerprint plot of the total (*a*) (top left) and specific (*b*) O⋯H/H⋯O (top right), (*c*) H⋯H,(bottom left) and (*d*) C⋯H/H⋯C (bottom right) inter­actions in [Ni_3_(C_8_H_4_O_4_)_3_(C_3_H_7_NO)_4_]. Note that H⋯H inter­actions coming from the methyl residues of DMF ligands are dominant and define the most relevant inter­actions among consecutive layers of the compound.

In order to visualize the inter­planar inter­actions, Hirshfeld surface (HS) analysis (Hirshfeld, 1977 ▸; Spackman & Jayatilaka, 2009 ▸) was performed by using *Crystal Explorer 17.5* (Turner *et al.*, 2017 ▸). In the HS plotted over *d*_norm_ (Fig. 5 ▸), the white surfaces indicate contacts with distances equal to the sum of van der Waals radii, and the red and blue colours indicate distances shorter (in close contact) or longer (distant contact) than the van der Waals radii, respectively (Venkatesan *et al.*, 2016 ▸). Since bonds from Ni1 to O atoms and from C2*B* and C4*B* to C atoms are not included in the asymmetric unit, bright-red spots appear over them. The following stronger short contacts shown as light-red spots correspond to weak C—H⋯O hydrogen bonds shown in Table 1 ▸. It is inter­esting to note that the ordered DMF-*C* mol­ecule shows one intra­molecular C1*C*—H1*C*⋯O1*A* and one inter­planar C2*C*—H2C*B*⋯O1*B* ^i^ hydrogen bond [symmetry code: (i) −*x* + 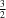, *y* + 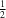, −*z* + 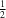]. The former limits the rotation of the DMF group and the latter the orientation. This fixes the DMF-*C* mol­ecules and provides the main inter­action between parallel network planes. The DMF-*D* mol­ecule, disordered over three positions, participates in no hydrogen bonds to the aldehyde carbon (C1*D*, C1*AD* or C1*BD*) but only to methyl H atoms, giving the mol­ecule rotational freedom. Additionally, the DMF mol­ecule is smaller than the void in which it sits, allowing for additional positional freedom. Removing DMF-*C* and DMF-*D* from the structural model, allowed the volume these mol­ecules occupy in the crystal structure to be calculated. The void-calculation routine in *PLATON* (Spek, 2009 ▸) was used, with a probe radius of 1.2 Å (enough to place small monoatomic ions). Voids arising from removing DMF-*C* and DMF-*D* are 110.18 and 167.93 Å^3^ per mol­ecule, respectively (two of the voids are connected around 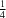, −0.07, 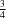 and 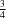, −0.02, 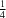 for DMF-*C* and 1/2,0.003,0 and 0,0.496,1/2 for DMF-*D*), again showing that the DMF-*D* mol­ecule is located over a much larger void than its own size, justifying the observation of positional disorder. Moreover, performing the same void calculation procedure using each of the DMF-*D* positions individually (as is the real case for each appearance of the mol­ecule in the crystal), it is observed that the highest occupied position of DMF-*D* leaves only 21.75 Å^3^ free volume per mol­ecule (in two smaller 10.88 Å^3^ voids) while DMF-*AD* and DMF-*BD* leave larger 53.1 and 37.7 Å^3^ voids, respectively. Besides the described hydrogen-bond inter­actions, contacts between H atoms from both DMF mol­ecules and neighbouring H, O and C atoms from surrounding DMF and tp anions dominate the inter­actions in the crystal structure, as depicted in Fig. 6 ▸, where the two-dimensional fingerprint plots (McKinnon *et al.*, 2007 ▸) are shown. H⋯H inter­actions from the DMF ligands are the most relevant, covering 45% of the Hirshfeld surface The presence of voids and a significant number of weak inter­layer inter­actions may well explain the possibility of using this material for Li-ion batteries, as will be discussed elsewhere.

## Database survey   

The May 2019 update of the CSD (Groom *et al.*, 2016 ▸) contains six coordination networks comprising Ni and a terephthalate anion as the sole linker; however, none of them contains only O in the coordination sphere. Additionally, there are eight trinuclear linear Ni compounds formed by carboxyl­ates and other oxygenated ligands, none of them coordination networks except for DAFHID (Chen *et al.*, 2011 ▸), which is discussed above.

## Synthesis and crystallization   

The compound was synthesized by solvothermal method *via* reaction between NiCl_2_·6H_2_O (0.6143 g, 2.58 mmol), terephthalic acid (0.8587 g, 5.20 mmol) and *N*,*N*-di­methyl­formamide (DMF)(50 ml) as a solvent; the reactants were dissolved in DMF and transferred to a steel autoclave at 423 K for 24 h.

The green crystals were collected by filtration, washed several times with DMF and dried at 373 K (yield 0.7 mg, 70%). Elemental Analysis for Ni_3_(C_8_H_4_O_4_)_3_(C_3_H_7_NO)_4_ (*M*_r_ = 960.81). Calculated (%): C 45.00, H 4.20, N 5.83, Ni 18.33. Found: C44.95, H 4.21, N 5.85, Ni 18.22.

## Refinement   

Crystal data, data collection and structure refinement details are summarized in Table 2 ▸. All hydrogen atoms were placed at geometrically suitable positions and refined riding with *U*_iso_(H) = 1.2 or 1.5 times the *U*_eq_ of the parent C atom. There are two sites occupied with *N*,*N*-di­methyl­formamide (DMF) mol­ecules; one of them showing disorder that was modelled in three different positions with relative occupancies of 0.502, 0.286 and 0.212. This causes C atoms from the DMF methyl groups to have very large thermal displacement parameters that required the use of similarity restraints to converge to reasonable values.

## Supplementary Material

Crystal structure: contains datablock(s) I. DOI: 10.1107/S2056989019014658/ex2025sup1.cif

Structure factors: contains datablock(s) I. DOI: 10.1107/S2056989019014658/ex2025Isup2.hkl

CCDC references: 1962320, 1962320

Additional supporting information:  crystallographic information; 3D view; checkCIF report

## Figures and Tables

**Table 1:** Hydrogen-bond geometry (Å, °)

*D*—H⋯*A*	*D*—H	H⋯*A*	*D*⋯*A*	*D*—H⋯*A*
C1*C*—H1*C*⋯O1*A*	0.93	2.33	2.893 (3)	119
C2*C*—H2*CB*⋯O1*B* ^i^	0.96	2.56	3.367 (5)	143
C1*AD*—H1*AD*⋯O2*B*	0.93	2.49	3.013 (7)	116
C2*AD*—H2*DE*⋯O1*B* ^ii^	0.96	2.39	3.089 (8)	130
C2*BD*—H2*DG*⋯O1*B* ^ii^	0.96	2.39	2.981 (10)	119

Symmetry codes: (i) 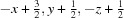; (ii) 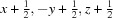.

**Table 2:** Experimental details

Crystal data	
Chemical formula	[Ni_3_(C_8_H_4_O_4_)_3_(C_3_H_7_NO)_4_]
*M*_r_	960.85
Crystal system, space group	Monoclinic, *P*2_1_/*n*
Temperature (K)	298
*a*, *b*, *c* (Å)	14.0309 (16), 9.6335 (11), 16.5804 (19)
β (°)	109.230 (5)
*V* (Å^3^)	2116.1 (4)
*Z*	2
Radiation type	Cu *K*α
μ (mm^−1^)	2.18
Crystal size (mm)	0.18 × 0.14 × 0.08

Data collection	
Diffractometer	Bruker D8 Venture
Absorption correction	Multi-scan (*SADABS*; Krause *et al.*, 2015 ▸)
*T*_min_, *T*_max_	0.657, 0.754
No. of measured, independent and observed [*I* > 2σ(*I*)] reflections	21976, 4170, 3539
*R*_int_	0.040
(sin θ/λ)_max_ (Å^−1^)	0.619

Refinement	
*R*[*F* ^2^ > 2σ(*F* ^2^)], *wR*(*F* ^2^), *S*	0.033, 0.092, 1.04
No. of reflections	4170
No. of parameters	339
No. of restraints	353
H-atom treatment	H-atom parameters constrained
Δρ_max_, Δρ_min_ (e Å^−3^)	0.28, −0.52

Computer programs: *APEX2* and *SAINT* (Bruker, 2014 ▸), *SHELXT* (Sheldrick, 2015*a* ▸), *SHELXL2018/3* (Sheldrick, 2015*b* ▸), *Mercury* (Macrae *et al.*, 2008 ▸), *VESTA* (Momma & Izumi, 2011 ▸) and *publCIF* (Westrip, 2010 ▸).

## supplementary crystallographic information

### Crystal data

**Table:**

[Ni_3_(C_8_H_4_O_4_)_3_(C_3_H_7_NO)_4_]	*F*(000) = 992
*M**_r_* = 960.85	*D*_x_ = 1.508 Mg m^−^^3^
Monoclinic, *P*2_1_/*n*	Cu *K*α radiation, λ = 1.54178 Å
*a* = 14.0309 (16) Å	Cell parameters from 9446 reflections
*b* = 9.6335 (11) Å	θ = 3.6–72.4°
*c* = 16.5804 (19) Å	µ = 2.18 mm^−^^1^
β = 109.230 (5)°	*T* = 298 K
*V* = 2116.1 (4) Å^3^	Plate, green
*Z* = 2	0.18 × 0.14 × 0.08 mm

### Data collection

**Table:**

Bruker D8 Venture diffractometer	4170 independent reflections
Radiation source: Incoatec microsource	3539 reflections with *I* > 2σ(*I*)
Detector resolution: 10.25 pixels mm^-1^	*R*_int_ = 0.040
/j and /w scans	θ_max_ = 72.5°, θ_min_ = 3.6°
Absorption correction: multi-scan (SADABS; Krause, *et al.*, 2015)	*h* = −16→17
*T*_min_ = 0.657, *T*_max_ = 0.754	*k* = −11→9
21976 measured reflections	*l* = −20→20

### Refinement

**Table:**

Refinement on *F*^2^	353 restraints
Least-squares matrix: full	Hydrogen site location: inferred from neighbouring sites
*R*[*F*^2^ > 2σ(*F*^2^)] = 0.033	H-atom parameters constrained
*wR*(*F*^2^) = 0.092	*w* = 1/[σ^2^(*F*_o_^2^) + (0.0499*P*)^2^ + 0.8233*P*] where *P* = (*F*_o_^2^ + 2*F*_c_^2^)/3
*S* = 1.04	(Δ/σ)_max_ = 0.001
4170 reflections	Δρ_max_ = 0.28 e Å^−^^3^
339 parameters	Δρ_min_ = −0.52 e Å^−^^3^

### Special details

**Table:**

Geometry. All esds (except the esd in the dihedral angle between two l.s. planes) are estimated using the full covariance matrix. The cell esds are taken into account individually in the estimation of esds in distances, angles and torsion angles; correlations between esds in cell parameters are only used when they are defined by crystal symmetry. An approximate (isotropic) treatment of cell esds is used for estimating esds involving l.s. planes.######################### VOID CALCULATED AFTER REMOVAL OF DMF-C (FROM PLATON) ========================================================================== Search for and Analysis of Solvent Accessible Voids in the Structure - Grid = 0.20 Ang., Probe Radius = 1.20 Ang., NStep = 6 ===============================================================================:: Total Potential Solvent Area Vol 440.3 Ang**3 per Unit Cell Vol 2116.1 Ang**3 [20.8%]Area #GridPoint VolPerc. Vol(A**3) X(av) Y(av) Z(av) Eigenvector(frac) Sig(Ang) ——————————————————————————- 1 30203[ 5658] 10 220[ 41.2] 0.250-0.068 0.750 1 0.125 1.000-0.231 3.00 2 0.245 0.677 1.000 1.85 3 1.000-0.310 0.187 1.20 2 30205[ 5658] 10 220[ 41.2] 0.750-0.018 0.250 1 0.158 1.000 0.025 2.91 2 -0.042 0.068 1.000 2.08 3 1.000-0.297 0.312 1.17 x y z Shortest Contacts within 4.5 Ang. (Excl. H) =============================================================================== 1 0.250-0.068 0.750 C2D 2.97; O1A 3.18; Ni2 3.31; O1B 3.66; O3A 3.72; C3BD 3.99; C2BD 4.06; C3A 4.29; O1AD 4.30; 2 0.750-0.018 0.250 Ni2 3.06; O1A 3.19; C2D 3.23; O3A 3.86; O1AD 3.89; O1D 4.02; O1BD 4.07; O1B 4.07; C2BD 4.31;######################### VOID CALCULATED AFTER REMOVAL OF DMF-D (FROM PLATON) ============================================================================== Search for and Analysis of Solvent Accessible Voids in the Structure - Grid = 0.20 Ang., Probe Radius = 1.20 Ang., NStep = 6 ===============================================================================:: Total Potential Solvent Area Vol 671.7 Ang**3 per Unit Cell Vol 2116.1 Ang**3 [31.7%]Area #GridPoint VolPerc. Vol(A**3) X(av) Y(av) Z(av) Eigenvector(frac) Sig(Ang) ——————————————————————————- 1 46072[ 11117] 16 336[ 81.0] 0.500 0.003 0.000 1 -0.393 1.000 0.491 3.56 2 0.059-1.000 0.603 1.59 3 1.000 0.423 0.458 1.49 2 46084[ 11117] 16 336[ 81.0] 1.000 0.496 0.500 1 0.393 1.000-0.490 3.56 2 0.037 1.000 0.589 1.59 3 1.000-0.401 0.471 1.49 x y z Shortest Contacts within 4.5 Ang. (Excl. H) =============================================================================== 1 0.500 0.003 0.000 O3A 3.66; C4A 3.72; O1C 3.86; Ni2 4.37; C3A 4.46; 2 1.000 0.496 0.500 O3A 3.66; C4A 3.72; O1C 3.86; Ni2 4.37; C3A 4.46;######################### VOID AROUND DMF-D (EXCLUDING DISORDERED POSITIONS AD AND BD) :: Total Potential Solvent Area Vol 43.5 Ang**3 per Unit Cell Vol 2116.1 Ang**3 [ 2.1%]Area #GridPoint VolPerc. Vol(A**3) X(av) Y(av) Z(av) Eigenvector(frac) Sig(Ang) ——————————————————————————- 1 1492[ 5] 1 11[ 0.0] 0.368 0.018 0.206 1 -0.079-0.096 1.000 0.63 2 0.359 1.000 0.150 0.62 3 -1.000 0.684-0.306 0.59 2 1492[ 5] 1 11[ 0.0] 0.632-0.018 0.794 1 -0.082-0.079 1.000 0.63 2 0.358 1.000 0.144 0.62 3 -1.000 0.681-0.312 0.59 3 1492[ 5] 1 11[ 0.0] 0.868 0.482 0.706 1 -0.062 0.034 1.000 0.63 2 -0.381 1.000-0.132 0.62 3 -1.000-0.722-0.310 0.59 4 1491[ 5] 1 11[ 0.0] 0.132 0.518 0.294 1 -0.038-0.048 1.000 0.63 2 -0.394 1.000-0.103 0.62 3 -1.000-0.744-0.315 0.59 x y z Shortest Contacts within 4.5 Ang. (Excl. H) =============================================================================== 1 0.368 0.018 0.206 N1D 2.91; C5A 3.04; C4A 3.14; C3D 3.23; C7A 3.24; C2D 3.25; C1D 3.30; C6A 3.31; C4B 3.33; 2 0.632-0.018 0.794 N1D 2.91; C5A 3.04; C4A 3.15; C3D 3.23; C7A 3.24; C2D 3.24; C1D 3.30; C6A 3.31; C4B 3.33; 3 0.868 0.482 0.706 N1D 2.91; C5A 3.04; C4A 3.15; C3D 3.23; C7A 3.23; C2D 3.25; C1D 3.30; C6A 3.31; C4B 3.33; =========================================================================== 4 0.132 0.518 0.294 N1D 2.91; C5A 3.04; C4A 3.14; C3D 3.23; C7A 3.24; C2D 3.25; C1D 3.30; C6A 3.31; C4B 3.33;######################### VOIDS AROUND DMF-AD (EXCLUDING DISORDERED POSITIONS D AND BD) :: Total Potential Solvent Area Vol 106.2 Ang**3 per Unit Cell Vol 2116.1 Ang**3 [ 5.0%]Area #GridPoint VolPerc. Vol(A**3) X(av) Y(av) Z(av) Eigenvector(frac) Sig(Ang) ——————————————————————————- 1 5282[ 133] 2 39[ 1.0] 0.500 0.000 1.000 1 0.349-0.255 1.000 1.55 2 1.000-0.019 0.030 0.81 3 0.043 1.000 0.097 0.68 2 998[ 1] 0 7[ 0.0] 0.750 0.042 0.571 1 1.000-0.023-0.164 0.55 2 0.580-0.051 1.000 0.54 3 -0.020-1.000-0.017 0.52 3 5291[ 133] 2 39[ 1.0] 1.000 0.500 0.500 1 0.349 0.255 1.000 1.55 2 1.000 0.007 0.031 0.81 3 -0.037 1.000-0.097 0.68 4 998[ 1] 0 7[ 0.0] 0.250 0.458 0.071 1 1.000-0.017-0.170 0.55 2 0.587-0.046 1.000 0.54 3 -0.017-1.000-0.016 0.52 5 998[ 1] 0 7[ 0.0] 0.750 0.542 0.929 1 1.000-0.032-0.157 0.55 2 0.571-0.119 1.000 0.54 3 -0.038-1.000-0.042 0.52 6 998[ 1] 0 7[ 0.0] 0.250 0.958 0.429 1 1.000-0.024-0.169 0.55 2 0.587-0.002 1.000 0.54 3 -0.010-1.000 0.001 0.52 =============================================================================== x y z Shortest Contacts within 4.5 Ang. (Excl. H) =============================================================================== 1 0.500 0.000 1.000 C3AD 3.20; O1AD 3.26; N1AD 3.29; C1AD 3.37; O3A 3.66; C4A 3.74; O1C 3.87; C2AD 4.26; Ni2 4.37; 2 0.750 0.042 0.571 C3B 2.91; C1AD 2.92; C6A 2.98; C2B 3.02; C2C 3.02; C5A 3.03; N1AD 3.08; C7A 3.13; C4A 3.21; 3 1.000 0.500 0.500 C3AD 3.20; O1AD 3.26; N1AD 3.29; C1AD 3.37; O3A 3.66; C4A 3.74; O1C 3.87; C2AD 4.26; Ni2 4.37; 4 0.250 0.458 0.071 C3B 2.91; C1AD 2.91; C6A 2.98; C2B 3.02; C2C 3.02; C5A 3.03; N1AD 3.08; C7A 3.13; C4A 3.21; 5 0.750 0.542 0.929 C3B 2.91; C1AD 2.92; C6A 2.98; C2B 3.02; C2C 3.02; C5A 3.03; N1AD 3.08; C7A 3.13; C4A 3.21; 6 0.250 0.958 0.429 C3B 2.91; C1AD 2.91; C6A 2.98; C2B 3.02; C2C 3.02; C5A 3.03; N1AD 3.07; C7A 3.13; C4A 3.21;######################### VOIDS AROUND DMF-BD (EXCLUDING DISORDERED POSITIONS D AND BD) :: Total Potential Solvent Area Vol 75.4 Ang**3 per Unit Cell Vol 2116.1 Ang**3 [ 3.6%]Area #GridPoint VolPerc. Vol(A**3) X(av) Y(av) Z(av) Eigenvector(frac) Sig(Ang) ——————————————————————————- 1 2589[ 15] 1 19[ 0.1] 0.766 0.088 0.618 1 -0.145 0.973 1.000 1.05 2 1.000 0.224 0.297 0.66 3 -0.110 1.000-0.356 0.60 2 2584[ 15] 1 19[ 0.1] 0.266 0.412 0.118 1 -0.139-0.965 1.000 1.05 2 1.000-0.242 0.289 0.66 3 0.123 1.000 0.358 0.59 3 2588[ 15] 1 19[ 0.1] 0.734 0.588 0.882 1 -0.143-0.973 1.000 1.05 2 1.000-0.230 0.294 0.66 3 0.114 1.000 0.358 0.60 4 2585[ 15] 1 19[ 0.1] 0.234 0.912 0.382 1 -0.147 0.967 1.000 1.05 2 1.000 0.207 0.304 0.66 3 -0.097 1.000-0.349 0.60 x y z Shortest Contacts within 4.5 Ang. (Excl. H) =============================================================================== 1 0.766 0.088 0.618 C7A 2.93; C1BD 2.93; C3B 2.97; C2A 3.09; C6A 3.13; N1BD 3.18; O1BD 3.21; C2B 3.38; C3A 3.40; 2 0.266 0.412 0.118 C1BD 2.93; C7A 2.93; C3B 2.97; C2A 3.09; C6A 3.13; N1BD 3.17; O1BD 3.21; C2B 3.38; C3A 3.41; 3 0.734 0.588 0.882 C7A 2.93; C1BD 2.93; C3B 2.97; C2A 3.09; C6A 3.13; N1BD 3.17; O1BD 3.21; C2B 3.38; C3A 3.40; 4 0.234 0.912 0.382 C1BD 2.93; C7A 2.93; C3B 2.97; C2A 3.09; C6A 3.13; N1BD 3.17; O1BD 3.21; C2B 3.39; C3A 3.41;

### Fractional atomic coordinates and isotropic or equivalent isotropic displacement parameters (Å^2^)

**Table:**

	*x*	*y*	*z*	*U*_iso_*/*U*_eq_	Occ. (<1)
Ni1	0.500000	0.500000	0.500000	0.02455 (12)	
Ni2	0.67419 (2)	0.42716 (4)	0.40049 (2)	0.03009 (11)	
O1A	0.56051 (11)	0.52816 (17)	0.31290 (9)	0.0386 (3)	
C1A	0.47483 (15)	0.5613 (2)	0.31483 (12)	0.0318 (4)	
O2A	0.44086 (11)	0.54372 (18)	0.37410 (9)	0.0406 (4)	
C2A	0.40430 (15)	0.6317 (2)	0.23606 (13)	0.0350 (4)	
O3A	0.23759 (11)	0.90569 (16)	−0.03005 (9)	0.0380 (3)	
C3A	0.43627 (17)	0.6769 (3)	0.17032 (14)	0.0453 (6)	
H3A	0.502355	0.659985	0.172565	0.054*	
O4A	0.11253 (10)	0.85882 (16)	0.02142 (10)	0.0384 (3)	
C3B	0.54232 (19)	0.1105 (2)	0.55325 (15)	0.0435 (5)	
H3B	0.570430	0.184531	0.589193	0.052*	
C5A	0.27238 (15)	0.7701 (2)	0.09543 (13)	0.0340 (4)	
C6A	0.23995 (18)	0.7236 (3)	0.16061 (16)	0.0550 (7)	
H6A	0.173356	0.738276	0.157606	0.066*	
C7A	0.30498 (18)	0.6555 (3)	0.23020 (16)	0.0568 (7)	
H7A	0.281846	0.625248	0.273702	0.068*	
C8A	0.20106 (15)	0.8513 (2)	0.02281 (12)	0.0315 (4)	
C4A	0.37147 (17)	0.7469 (3)	0.10111 (14)	0.0445 (6)	
H4A	0.394827	0.778659	0.058044	0.053*	
O1B	0.59907 (13)	0.22615 (17)	0.36814 (10)	0.0447 (4)	
C1B	0.57649 (15)	0.2365 (2)	0.43443 (14)	0.0349 (4)	
O2B	0.59547 (10)	0.35001 (14)	0.47735 (9)	0.0323 (3)	
C2B	0.53512 (16)	0.1155 (2)	0.46820 (15)	0.0364 (5)	
C4B	0.50778 (19)	−0.0046 (2)	0.58491 (15)	0.0438 (5)	
H4B	0.513277	−0.007863	0.642327	0.053*	
O1C	0.75142 (13)	0.4275 (2)	0.31558 (11)	0.0528 (4)	
C1C	0.7187 (2)	0.4691 (3)	0.24174 (18)	0.0558 (6)	
H1C	0.664285	0.529957	0.228484	0.067*	
N1C	0.75234 (19)	0.4370 (3)	0.18062 (15)	0.0567 (6)	
C2C	0.7109 (3)	0.4958 (5)	0.0954 (2)	0.0936 (12)	
H2CA	0.682464	0.422950	0.055091	0.140*	
H2CB	0.763583	0.541335	0.080424	0.140*	
H2CC	0.659234	0.561783	0.094316	0.140*	
C3C	0.8336 (4)	0.3417 (6)	0.1934 (3)	0.1189 (17)	
H3CA	0.880182	0.351911	0.250325	0.178*	
H3CB	0.867587	0.360371	0.152930	0.178*	
H3CC	0.807666	0.248664	0.185574	0.178*	
O1D	0.8014 (9)	0.3151 (12)	0.4826 (6)	0.0530 (6)	0.502 (2)
C1D	0.8794 (4)	0.3625 (6)	0.5229 (3)	0.0571 (10)	0.502 (2)
H1D	0.897601	0.443924	0.501510	0.069*	0.502 (2)
N1D	0.9445 (4)	0.3160 (7)	0.5941 (3)	0.0713 (9)	0.502 (2)
C2D	1.0427 (5)	0.3755 (9)	0.6322 (5)	0.101 (2)	0.502 (2)
H2DA	1.040810	0.440932	0.675326	0.151*	0.502 (2)
H2DB	1.062711	0.422124	0.589159	0.151*	0.502 (2)
H2DC	1.090317	0.303414	0.657648	0.151*	0.502 (2)
C3D	0.9207 (6)	0.1915 (9)	0.6338 (5)	0.109 (2)	0.502 (2)
H3DA	0.892358	0.122527	0.590807	0.164*	0.502 (2)
H3DB	0.872814	0.214163	0.661918	0.164*	0.502 (2)
H3DC	0.981193	0.155916	0.674938	0.164*	0.502 (2)
O1AD	0.7959 (15)	0.317 (4)	0.4743 (6)	0.0530 (6)	0.285 (3)
C1AD	0.8215 (6)	0.3181 (12)	0.5491 (5)	0.0587 (12)	0.285 (3)
H1AD	0.774908	0.353722	0.572603	0.070*	0.285 (3)
N1AD	0.9073 (5)	0.2759 (10)	0.6039 (4)	0.0713 (9)	0.285 (3)
C2AD	0.9272 (8)	0.2782 (15)	0.6944 (4)	0.085 (3)	0.285 (3)
H2DD	0.920908	0.371541	0.712322	0.127*	0.285 (3)
H2DE	0.994468	0.245201	0.722992	0.127*	0.285 (3)
H2DF	0.879667	0.219575	0.708430	0.127*	0.285 (3)
C3AD	0.9776 (7)	0.1978 (14)	0.5730 (6)	0.090 (3)	0.285 (3)
H3DD	0.941746	0.125546	0.535186	0.135*	0.285 (3)
H3DE	1.029120	0.157344	0.620566	0.135*	0.285 (3)
H3DF	1.008036	0.259050	0.542830	0.135*	0.285 (3)
O1BD	0.7929 (14)	0.315 (4)	0.4838 (9)	0.0530 (6)	0.213 (3)
C1BD	0.8318 (6)	0.3434 (18)	0.5559 (6)	0.0600 (13)	0.213 (3)
H1BD	0.792321	0.392423	0.581459	0.072*	0.213 (3)
N1BD	0.9240 (5)	0.3147 (15)	0.6053 (5)	0.0713 (9)	0.213 (3)
C2BD	0.9529 (10)	0.343 (2)	0.6968 (5)	0.087 (3)	0.213 (3)
H2DG	1.025064	0.340840	0.721607	0.131*	0.213 (3)
H2DH	0.924006	0.273742	0.723433	0.131*	0.213 (3)
H2DI	0.928462	0.432967	0.705483	0.131*	0.213 (3)
C3BD	1.0039 (7)	0.2844 (19)	0.5720 (7)	0.081 (3)	0.213 (3)
H3DG	0.976030	0.268049	0.511667	0.122*	0.213 (3)
H3DH	1.039618	0.203269	0.599686	0.122*	0.213 (3)
H3DI	1.049555	0.361718	0.582424	0.122*	0.213 (3)

### Atomic displacement parameters (Å^2^)

**Table:**

	*U*^11^	*U*^22^	*U*^33^	*U*^12^	*U*^13^	*U*^23^
Ni1	0.0235 (2)	0.0262 (2)	0.0194 (2)	−0.00175 (17)	0.00091 (16)	0.00035 (17)
Ni2	0.02733 (18)	0.0330 (2)	0.02504 (18)	0.00005 (13)	0.00201 (13)	−0.00149 (14)
O1A	0.0326 (7)	0.0499 (9)	0.0283 (7)	0.0057 (7)	0.0033 (6)	0.0066 (7)
C1A	0.0318 (10)	0.0330 (10)	0.0252 (9)	−0.0013 (8)	0.0023 (8)	−0.0015 (8)
O2A	0.0353 (8)	0.0580 (10)	0.0237 (7)	0.0064 (7)	0.0032 (6)	0.0076 (7)
C2A	0.0322 (10)	0.0405 (11)	0.0268 (9)	0.0038 (9)	0.0024 (8)	0.0043 (9)
O3A	0.0362 (7)	0.0418 (8)	0.0330 (7)	0.0109 (6)	0.0074 (6)	0.0090 (6)
C3A	0.0301 (10)	0.0671 (16)	0.0363 (11)	0.0127 (10)	0.0075 (9)	0.0136 (11)
O4A	0.0290 (7)	0.0351 (8)	0.0463 (8)	0.0070 (6)	0.0058 (6)	0.0090 (7)
C3B	0.0567 (14)	0.0279 (11)	0.0424 (12)	−0.0084 (10)	0.0115 (10)	−0.0070 (9)
C5A	0.0305 (10)	0.0384 (11)	0.0276 (9)	0.0065 (8)	0.0022 (8)	0.0034 (8)
C6A	0.0314 (11)	0.086 (2)	0.0464 (13)	0.0138 (12)	0.0116 (10)	0.0248 (14)
C7A	0.0384 (12)	0.090 (2)	0.0422 (13)	0.0134 (13)	0.0139 (10)	0.0298 (14)
C8A	0.0309 (10)	0.0291 (10)	0.0289 (9)	0.0042 (8)	0.0023 (8)	0.0002 (8)
C4A	0.0366 (11)	0.0638 (16)	0.0319 (11)	0.0110 (11)	0.0098 (9)	0.0157 (11)
O1B	0.0556 (9)	0.0363 (8)	0.0430 (9)	−0.0060 (7)	0.0174 (7)	−0.0057 (7)
C1B	0.0325 (10)	0.0291 (10)	0.0377 (11)	0.0017 (8)	0.0044 (8)	0.0000 (9)
O2B	0.0324 (7)	0.0250 (7)	0.0365 (7)	−0.0010 (6)	0.0074 (6)	−0.0015 (6)
C2B	0.0376 (11)	0.0252 (10)	0.0438 (11)	0.0010 (8)	0.0099 (9)	−0.0004 (9)
C4B	0.0583 (14)	0.0333 (12)	0.0385 (11)	−0.0052 (10)	0.0140 (10)	−0.0034 (10)
O1C	0.0464 (9)	0.0731 (12)	0.0419 (9)	0.0095 (8)	0.0188 (7)	0.0067 (9)
C1C	0.0560 (14)	0.0654 (16)	0.0487 (13)	0.0090 (12)	0.0208 (11)	0.0019 (12)
N1C	0.0714 (14)	0.0577 (13)	0.0486 (12)	−0.0040 (11)	0.0301 (11)	−0.0011 (10)
C2C	0.110 (3)	0.120 (3)	0.0534 (18)	−0.020 (3)	0.0299 (19)	0.011 (2)
C3C	0.149 (4)	0.127 (4)	0.109 (3)	0.053 (3)	0.080 (3)	0.012 (3)
O1D	0.0397 (12)	0.0572 (10)	0.0484 (10)	0.0110 (9)	−0.0040 (9)	0.0014 (14)
C1D	0.0406 (16)	0.0618 (18)	0.0543 (17)	0.0079 (16)	−0.0040 (15)	0.0058 (17)
N1D	0.0537 (15)	0.0745 (15)	0.0625 (13)	0.0094 (13)	−0.0124 (12)	0.0064 (13)
C2D	0.067 (3)	0.104 (4)	0.097 (4)	−0.001 (3)	−0.021 (3)	−0.001 (4)
C3D	0.094 (4)	0.106 (4)	0.093 (4)	−0.003 (3)	−0.015 (3)	0.022 (3)
O1AD	0.0397 (12)	0.0572 (10)	0.0484 (10)	0.0110 (9)	−0.0040 (9)	0.0014 (14)
C1AD	0.0454 (19)	0.065 (2)	0.0521 (19)	0.0110 (18)	−0.0028 (18)	0.004 (2)
N1AD	0.0537 (15)	0.0745 (15)	0.0625 (13)	0.0094 (13)	−0.0124 (12)	0.0064 (13)
C2AD	0.077 (4)	0.083 (4)	0.070 (4)	0.005 (4)	−0.009 (4)	0.009 (4)
C3AD	0.065 (4)	0.095 (4)	0.083 (4)	0.015 (4)	−0.011 (4)	0.004 (4)
O1BD	0.0397 (12)	0.0572 (10)	0.0484 (10)	0.0110 (9)	−0.0040 (9)	0.0014 (14)
C1BD	0.046 (2)	0.065 (2)	0.053 (2)	0.0109 (19)	−0.0046 (19)	0.004 (2)
N1BD	0.0537 (15)	0.0745 (15)	0.0625 (13)	0.0094 (13)	−0.0124 (12)	0.0064 (13)
C2BD	0.071 (4)	0.092 (4)	0.071 (4)	0.001 (4)	−0.013 (4)	0.007 (4)
C3BD	0.061 (4)	0.087 (4)	0.074 (4)	0.007 (4)	−0.008 (4)	0.003 (4)

### Geometric parameters (Å, º)

**Table:**

Ni1—O2A^i^	2.0205 (14)	N1C—C3C	1.424 (5)
Ni1—O2A	2.0206 (14)	N1C—C2C	1.454 (4)
Ni1—O4A^ii^	2.0246 (14)	C2C—H2CA	0.9600
Ni1—O4A^iii^	2.0246 (14)	C2C—H2CB	0.9600
Ni1—O2B^i^	2.0868 (14)	C2C—H2CC	0.9600
Ni1—O2B	2.0868 (14)	C3C—H3CA	0.9600
Ni2—O3A^ii^	2.0090 (15)	C3C—H3CB	0.9600
Ni2—O1A	2.0184 (15)	C3C—H3CC	0.9600
Ni2—O1C	2.0399 (17)	O1D—C1D	1.171 (10)
Ni2—O1AD	2.042 (12)	C1D—N1D	1.311 (4)
Ni2—O2B	2.0791 (15)	C1D—H1D	0.9300
Ni2—O1BD	2.081 (19)	N1D—C2D	1.432 (7)
Ni2—O1D	2.146 (6)	N1D—C3D	1.460 (8)
Ni2—O1B	2.1853 (16)	C2D—H2DA	0.9600
Ni2—C1B	2.465 (2)	C2D—H2DB	0.9600
O1A—C1A	1.255 (3)	C2D—H2DC	0.9600
C1A—O2A	1.237 (3)	C3D—H3DA	0.9600
C1A—C2A	1.514 (3)	C3D—H3DB	0.9600
C2A—C3A	1.379 (3)	C3D—H3DC	0.9600
C2A—C7A	1.384 (3)	O1AD—C1AD	1.171 (10)
O3A—C8A	1.266 (3)	C1AD—N1AD	1.312 (4)
C3A—C4A	1.383 (3)	C1AD—H1AD	0.9300
C3A—H3A	0.9300	N1AD—C2AD	1.433 (7)
O4A—C8A	1.237 (2)	N1AD—C3AD	1.461 (8)
C3B—C4B	1.381 (3)	C2AD—H2DD	0.9600
C3B—C2B	1.381 (3)	C2AD—H2DE	0.9600
C3B—H3B	0.9300	C2AD—H2DF	0.9600
C5A—C6A	1.378 (3)	C3AD—H3DD	0.9600
C5A—C4A	1.380 (3)	C3AD—H3DE	0.9600
C5A—C8A	1.506 (3)	C3AD—H3DF	0.9600
C6A—C7A	1.379 (3)	O1BD—C1BD	1.171 (10)
C6A—H6A	0.9300	C1BD—N1BD	1.312 (4)
C7A—H7A	0.9300	C1BD—H1BD	0.9300
C4A—H4A	0.9300	N1BD—C3BD	1.433 (7)
O1B—C1B	1.244 (3)	N1BD—C2BD	1.461 (8)
C1B—O2B	1.284 (3)	C2BD—H2DG	0.9600
C1B—C2B	1.491 (3)	C2BD—H2DH	0.9600
C2B—C4B^iv^	1.389 (3)	C2BD—H2DI	0.9600
C4B—H4B	0.9300	C3BD—H3DG	0.9600
O1C—C1C	1.225 (3)	C3BD—H3DH	0.9600
C1C—N1C	1.288 (4)	C3BD—H3DI	0.9600
C1C—H1C	0.9300		
			
O2A^i^—Ni1—O2A	180.0	C1B—O2B—Ni2	91.14 (13)
O2A^i^—Ni1—O4A^ii^	85.85 (7)	C1B—O2B—Ni1	131.39 (13)
O2A—Ni1—O4A^ii^	94.15 (7)	Ni2—O2B—Ni1	111.40 (6)
O2A^i^—Ni1—O4A^iii^	94.15 (7)	C3B—C2B—C4B^iv^	119.4 (2)
O2A—Ni1—O4A^iii^	85.85 (7)	C3B—C2B—C1B	120.3 (2)
O4A^ii^—Ni1—O4A^iii^	180.00 (12)	C4B^iv^—C2B—C1B	120.3 (2)
O2A^i^—Ni1—O2B^i^	91.60 (6)	C3B—C4B—C2B^iv^	120.6 (2)
O2A—Ni1—O2B^i^	88.40 (6)	C3B—C4B—H4B	119.7
O4A^ii^—Ni1—O2B^i^	90.75 (6)	C2B^iv^—C4B—H4B	119.7
O4A^iii^—Ni1—O2B^i^	89.25 (6)	C1C—O1C—Ni2	125.55 (17)
O2A^i^—Ni1—O2B	88.40 (6)	O1C—C1C—N1C	126.5 (3)
O2A—Ni1—O2B	91.60 (6)	O1C—C1C—H1C	116.8
O4A^ii^—Ni1—O2B	89.25 (6)	N1C—C1C—H1C	116.8
O4A^iii^—Ni1—O2B	90.75 (6)	C1C—N1C—C3C	121.3 (3)
O2B^i^—Ni1—O2B	180.00 (5)	C1C—N1C—C2C	122.6 (3)
O3A^ii^—Ni2—O1A	97.00 (7)	C3C—N1C—C2C	116.1 (3)
O3A^ii^—Ni2—O1C	99.13 (7)	N1C—C2C—H2CA	109.5
O1A—Ni2—O1C	88.63 (7)	N1C—C2C—H2CB	109.5
O3A^ii^—Ni2—O1AD	87.1 (9)	H2CA—C2C—H2CB	109.5
O1A—Ni2—O1AD	171.6 (5)	N1C—C2C—H2CC	109.5
O1C—Ni2—O1AD	83.4 (7)	H2CA—C2C—H2CC	109.5
O3A^ii^—Ni2—O2B	99.18 (6)	H2CB—C2C—H2CC	109.5
O1A—Ni2—O2B	99.20 (6)	N1C—C3C—H3CA	109.5
O1C—Ni2—O2B	159.02 (7)	N1C—C3C—H3CB	109.5
O1AD—Ni2—O2B	87.4 (9)	H3CA—C3C—H3CB	109.5
O3A^ii^—Ni2—O1BD	85.9 (9)	N1C—C3C—H3CC	109.5
O1A—Ni2—O1BD	176.0 (6)	H3CA—C3C—H3CC	109.5
O1C—Ni2—O1BD	88.2 (7)	H3CB—C3C—H3CC	109.5
O2B—Ni2—O1BD	83.0 (9)	C1D—O1D—Ni2	126.2 (8)
O3A^ii^—Ni2—O1D	85.5 (3)	O1D—C1D—N1D	128.5 (7)
O1A—Ni2—O1D	173.8 (4)	O1D—C1D—H1D	115.7
O1C—Ni2—O1D	85.3 (5)	N1D—C1D—H1D	115.7
O2B—Ni2—O1D	86.0 (5)	C1D—N1D—C2D	123.0 (5)
O3A^ii^—Ni2—O1B	159.66 (6)	C1D—N1D—C3D	119.5 (5)
O1A—Ni2—O1B	92.73 (7)	C2D—N1D—C3D	117.4 (4)
O1C—Ni2—O1B	98.91 (7)	N1D—C2D—H2DA	109.5
O1AD—Ni2—O1B	85.7 (10)	N1D—C2D—H2DB	109.5
O2B—Ni2—O1B	61.52 (6)	H2DA—C2D—H2DB	109.5
O1BD—Ni2—O1B	85.4 (10)	N1D—C2D—H2DC	109.5
O1D—Ni2—O1B	86.7 (4)	H2DA—C2D—H2DC	109.5
O3A^ii^—Ni2—C1B	129.86 (7)	H2DB—C2D—H2DC	109.5
O1A—Ni2—C1B	99.00 (7)	N1D—C3D—H3DA	109.5
O1C—Ni2—C1B	128.35 (8)	N1D—C3D—H3DB	109.5
O1AD—Ni2—C1B	83.8 (11)	H3DA—C3D—H3DB	109.5
O2B—Ni2—C1B	31.38 (6)	N1D—C3D—H3DC	109.5
O1BD—Ni2—C1B	81.1 (11)	H3DA—C3D—H3DC	109.5
O1D—Ni2—C1B	83.6 (5)	H3DB—C3D—H3DC	109.5
O1B—Ni2—C1B	30.28 (7)	C1AD—O1AD—Ni2	122.2 (11)
C1A—O1A—Ni2	129.89 (14)	O1AD—C1AD—N1AD	128.4 (7)
O2A—C1A—O1A	127.48 (18)	O1AD—C1AD—H1AD	115.8
O2A—C1A—C2A	115.67 (18)	N1AD—C1AD—H1AD	115.8
O1A—C1A—C2A	116.84 (18)	C1AD—N1AD—C2AD	122.6 (5)
C1A—O2A—Ni1	135.62 (14)	C1AD—N1AD—C3AD	119.2 (5)
C3A—C2A—C7A	118.32 (19)	C2AD—N1AD—C3AD	117.0 (4)
C3A—C2A—C1A	122.19 (19)	N1AD—C2AD—H2DD	109.5
C7A—C2A—C1A	119.46 (19)	N1AD—C2AD—H2DE	109.5
C8A—O3A—Ni2^v^	121.68 (13)	H2DD—C2AD—H2DE	109.5
C2A—C3A—C4A	120.9 (2)	N1AD—C2AD—H2DF	109.5
C2A—C3A—H3A	119.5	H2DD—C2AD—H2DF	109.5
C4A—C3A—H3A	119.5	H2DE—C2AD—H2DF	109.5
C8A—O4A—Ni1^vi^	139.74 (14)	N1AD—C3AD—H3DD	109.5
C4B—C3B—C2B	120.0 (2)	N1AD—C3AD—H3DE	109.5
C4B—C3B—H3B	120.0	H3DD—C3AD—H3DE	109.5
C2B—C3B—H3B	120.0	N1AD—C3AD—H3DF	109.5
C6A—C5A—C4A	118.62 (19)	H3DD—C3AD—H3DF	109.5
C6A—C5A—C8A	119.45 (19)	H3DE—C3AD—H3DF	109.5
C4A—C5A—C8A	121.84 (19)	C1BD—O1BD—Ni2	124.0 (19)
C5A—C6A—C7A	120.8 (2)	O1BD—C1BD—N1BD	128.4 (7)
C5A—C6A—H6A	119.6	O1BD—C1BD—H1BD	115.8
C7A—C6A—H6A	119.6	N1BD—C1BD—H1BD	115.8
C6A—C7A—C2A	120.8 (2)	C1BD—N1BD—C3BD	122.5 (5)
C6A—C7A—H7A	119.6	C1BD—N1BD—C2BD	119.2 (5)
C2A—C7A—H7A	119.6	C3BD—N1BD—C2BD	117.0 (4)
O4A—C8A—O3A	126.59 (18)	N1BD—C2BD—H2DG	109.5
O4A—C8A—C5A	116.37 (18)	N1BD—C2BD—H2DH	109.5
O3A—C8A—C5A	117.04 (18)	H2DG—C2BD—H2DH	109.5
C5A—C4A—C3A	120.5 (2)	N1BD—C2BD—H2DI	109.5
C5A—C4A—H4A	119.7	H2DG—C2BD—H2DI	109.5
C3A—C4A—H4A	119.7	H2DH—C2BD—H2DI	109.5
C1B—O1B—Ni2	87.41 (13)	N1BD—C3BD—H3DG	109.5
O1B—C1B—O2B	119.4 (2)	N1BD—C3BD—H3DH	109.5
O1B—C1B—C2B	120.87 (19)	H3DG—C3BD—H3DH	109.5
O2B—C1B—C2B	119.55 (19)	N1BD—C3BD—H3DI	109.5
O1B—C1B—Ni2	62.32 (12)	H3DG—C3BD—H3DI	109.5
O2B—C1B—Ni2	57.48 (10)	H3DH—C3BD—H3DI	109.5
C2B—C1B—Ni2	169.32 (15)		
			
Ni2—O1A—C1A—O2A	3.0 (3)	Ni2—O1B—C1B—C2B	−168.31 (18)
Ni2—O1A—C1A—C2A	−177.23 (14)	O1B—C1B—O2B—Ni2	−7.3 (2)
O1A—C1A—O2A—Ni1	16.3 (4)	C2B—C1B—O2B—Ni2	168.02 (17)
C2A—C1A—O2A—Ni1	−163.41 (16)	O1B—C1B—O2B—Ni1	113.0 (2)
O2A—C1A—C2A—C3A	170.1 (2)	C2B—C1B—O2B—Ni1	−71.7 (2)
O1A—C1A—C2A—C3A	−9.7 (3)	Ni2—C1B—O2B—Ni1	120.27 (16)
O2A—C1A—C2A—C7A	−8.0 (3)	C4B—C3B—C2B—C4B^iv^	0.5 (4)
O1A—C1A—C2A—C7A	172.2 (2)	C4B—C3B—C2B—C1B	−177.2 (2)
C7A—C2A—C3A—C4A	1.4 (4)	O1B—C1B—C2B—C3B	157.8 (2)
C1A—C2A—C3A—C4A	−176.8 (2)	O2B—C1B—C2B—C3B	−17.4 (3)
C4A—C5A—C6A—C7A	0.1 (4)	Ni2—C1B—C2B—C3B	53.4 (9)
C8A—C5A—C6A—C7A	−176.5 (3)	O1B—C1B—C2B—C4B^iv^	−19.9 (3)
C5A—C6A—C7A—C2A	−0.3 (5)	O2B—C1B—C2B—C4B^iv^	164.9 (2)
C3A—C2A—C7A—C6A	−0.4 (5)	Ni2—C1B—C2B—C4B^iv^	−124.3 (8)
C1A—C2A—C7A—C6A	177.8 (3)	C2B—C3B—C4B—C2B^iv^	−0.5 (4)
Ni1^vi^—O4A—C8A—O3A	−42.2 (4)	Ni2—O1C—C1C—N1C	−159.3 (2)
Ni1^vi^—O4A—C8A—C5A	137.12 (18)	O1C—C1C—N1C—C3C	1.8 (6)
Ni2^v^—O3A—C8A—O4A	24.2 (3)	O1C—C1C—N1C—C2C	−177.7 (3)
Ni2^v^—O3A—C8A—C5A	−155.13 (14)	Ni2—O1D—C1D—N1D	153.1 (8)
C6A—C5A—C8A—O4A	−7.5 (3)	O1D—C1D—N1D—C2D	172.9 (11)
C4A—C5A—C8A—O4A	175.9 (2)	O1D—C1D—N1D—C3D	−2.2 (15)
C6A—C5A—C8A—O3A	171.9 (2)	Ni2—O1AD—C1AD—N1AD	−165.3 (13)
C4A—C5A—C8A—O3A	−4.7 (3)	O1AD—C1AD—N1AD—C2AD	−177 (3)
C6A—C5A—C4A—C3A	0.8 (4)	O1AD—C1AD—N1AD—C3AD	−10 (3)
C8A—C5A—C4A—C3A	177.4 (2)	Ni2—O1BD—C1BD—N1BD	−154.2 (15)
C2A—C3A—C4A—C5A	−1.6 (4)	O1BD—C1BD—N1BD—C3BD	22 (3)
Ni2—O1B—C1B—O2B	6.94 (19)	O1BD—C1BD—N1BD—C2BD	−172 (3)

Symmetry codes: (i) −*x*+1, −*y*+1, −*z*+1; (ii) *x*+1/2, −*y*+3/2, *z*+1/2; (iii) −*x*+1/2, *y*−1/2, −*z*+1/2; (iv) −*x*+1, −*y*, −*z*+1; (v) *x*−1/2, −*y*+3/2, *z*−1/2; (vi) −*x*+1/2, *y*+1/2, −*z*+1/2.

### Hydrogen-bond geometry (Å, º)

**Table:**

*D*—H···*A*	*D*—H	H···*A*	*D*···*A*	*D*—H···*A*
C1*C*—H1*C*···O1*A*	0.93	2.33	2.893 (3)	119
C2*C*—H2*CB*···O1*B*^vii^	0.96	2.56	3.367 (5)	143
C1*AD*—H1*AD*···O2*B*	0.93	2.49	3.013 (7)	116
C2*AD*—H2*DE*···O1*B*^viii^	0.96	2.39	3.089 (8)	130
C2*BD*—H2*DG*···O1*B*^viii^	0.96	2.39	2.981 (10)	119

Symmetry codes: (vii) −*x*+3/2, *y*+1/2, −*z*+1/2; (viii) *x*+1/2, −*y*+1/2, *z*+1/2.
